# A case of subcutaneous emphysema/mediastinal emphysema during the use of humidified high-flow nasal cannula

**DOI:** 10.1186/s40981-019-0305-3

**Published:** 2019-12-26

**Authors:** Shota Sonobe, Satoki Inoue, Tadashi Nishiwada, Junji Egawa, Masahiko Kawaguchi

**Affiliations:** 1 Division of Intensive Care, Nara Medical University, 840 Shijo-cho, Kashihara, Nara, 634-8522 Japan; 2 Department of Anesthesiology, Nara Medical University, Kashihara, Nara, Japan

**Keywords:** Humidified high-flow nasal cannula, Subcutaneous emphysema, Mediastinal emphysema, Pneumothorax, Respiratory failure

## Abstract

**Background:**

Heated, humidified, high-flow nasal cannula (HHFNC) oxygen therapy allows optimal humidification of inspired gas at high flows and creates a distending pressure similar to nasal continuous positive airway pressure [1]. It has been safely used in adults with moderate hypoxemia with few complications [2, 3]. Hereby, we report serious complications occurred during HHFNC oxygen therapy.

**Case presentation:**

A 53-year-old female with hemophagocytic lymphohistiocytosis (HLH) was admitted to the intensive care unit because of respiratory failure. After weaning from mechanical ventilation which lasted for 2 weeks, HHFNC therapy at 40 L/min with an FiO2 of 0.5 was started for hypoxemia. Four days later, dyspnea and hypoxemia occurred and chest X-ray and CT scan revealed localized pneumothorax, subcutaneous emphysema, and massive pneumomediastinum. After cessation of HHFNC, respiratory condition improved.

**Conclusion:**

Subcutaneous emphysema, pneumothorax, and pneumomediastinum should be notified as a serious complication during HHFNC therapy.

## Background

Humidified high-flow nasal cannula (HHFNC) is useful before and after mechanical ventilation in tracheal intubation. Above all, it has been considered to be of advantage that it can be done safely and that the patient’s QOL is maintained. There are other benefits as well. For example, the positive end-expiratory pressure (PEEP) effect of HHFNC is considered to have a positive effect on acute heart failure, and it is also said that it has a CO_2_ washout effect at high flow rates. Meanwhile, as an adverse effect, Kang and colleagues found that using HHFNC for longer than 48 h before intubation was associated with higher ICU mortality and less success at extubation and ventilator weaning, and fewer ventilator-free days. They examined all subjects who failed HHFNC delayed and required intubation [4]. In this time, we experienced patients with severe complications. This manuscript was contributed for the purpose of making it widely known and hoping that preventing such complications will ensure the safety of the patient.

## Case presentation

**Fig. 1:**
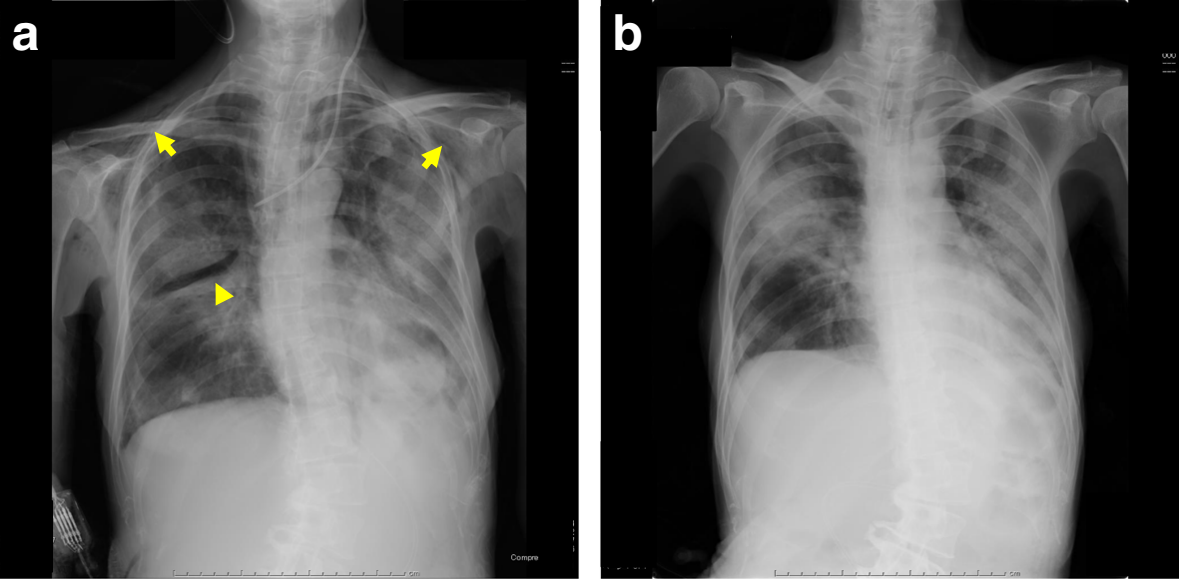
**a**, **b** Chest X ray **a** during and **b** 1 day after cessation of humidified, high-flow nasal cannula oxygen therapy. There is subcutaneous emphysema (arrow) and localized pneumothorax (arrowhead)

**Fig. 2:**
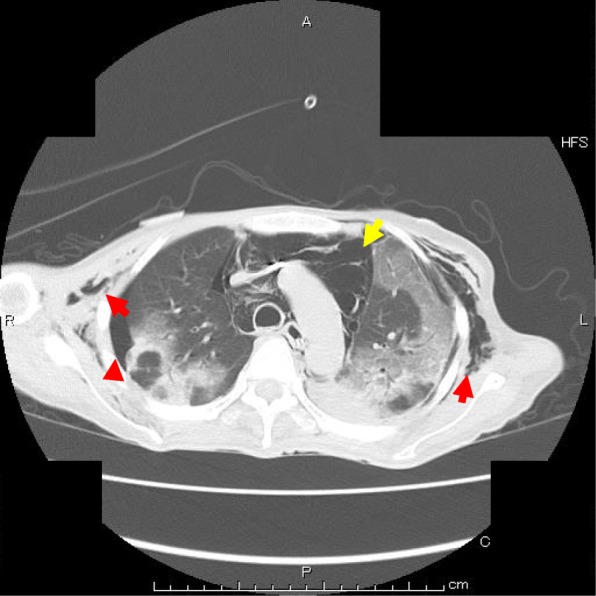
A subsequent chest CT following chest X-ray at deteriorated respiratory status. There are subcutaneous emphysema (red arrow), localized pneumothorax (red arrowhead), and pneumomediastinum (yellow arrow)

A 53-year-old female with hemophagocytic lymphohistiocytosis (HLH) was admitted to the intensive care unit (ICU) because of respiratory failure. Immediately after admission, her airway was established using a cuffed 7.0-mm tracheal tube. Her chest X-ray showed severe bilateral infiltrates, and blood gas showed moderate level of acute respiratory distress syndrome (ARDS). Immediately after beginning of mechanical ventilation, high positive end-expiratory pressure (PEEP) combined with low tidal volume was applied. Her respiratory status gradually improved. Two weeks after commencement of mechanical ventilation, finally, her trachea was extubated. However, she developed mild hypoxemia with standard oxygen therapy and was suffering from tenacious secretion. HHFNC therapy at 40 L/min with an F_I_O_2_ of 0.5 (Optiflow system™, MR850 heated humidified RT202 delivery tubing; Fisher and Paykel Healthcare Ltd., Auckland, New Zealand) was applied through nasal cannula (Optiflow™; Fisher and Paykel, Auckland, New Zealand), and a mini tracheostomy cannula (Portex™ Mini-Trach™ II, Smith Medical, Kent, UK) was placed to treat her sputum. Her oxygenation was improved, and F_I_O_2_ was decreased to 0.35. Four days later, she complained of respiratory discomfort. Her oxygenation worsened and hemodynamic status also deteriorated. Specifically, F_I_O_2_ was 0.35 and PaO_2_ was about 60 mmHg, and PaCO_2_ was less than 30 mmHg because of respiratory acceleration (35 per minute). In hemodynamics, blood pressure itself was maintained, but it became sinus tachycardia of about 100 to 130 times per minute. Her chest X-ray revealed a localized pneumothorax and subcutaneous emphysema (Fig. 1a). A subsequent chest CT showed massive pneumomediastinum and a localized right-side pneumothorax, and subcutaneous emphysema were confirmed (Fig. 2). Regarding these air leaks, they seemed difficult to release. We decided to observe her course with changing HHFNC therapy to the standard oxygen therapy (5 L/min of O_2_ administration through a standard oxygen mask). She could not improve subjective discomfort easily, but her oxygenation and hemodynamic status gradually improved. On the next day, it was confirmed by a chest X-ray that the localized pneumothorax and emphysema disappeared (Fig. 1b).

Moreover, she had a lung with bulla, and it is undeniable that the bulla ruptured due to cough caused by suction after extubation, and the pressure by HHFNC was applied to it and pneumothorax occurred.

## Discussion

The air leak is a well-known complication of positive pressure ventilation, and HHFNC provides increased pressure within airways [5], which may potentially cause air leak.

In the present case, air leak would have been ascribed to positive airway pressure generated by HHFNC through micro-tear of the trachea caused by mini-tracheostomy cannula placement or damage to the tracheal wall due to suctioning through the mini-tracheostomy. Otherwise, air leak might have occurred due to rupture of bulla and alveoli due to cough associated with suctioning. However, these could not go beyond speculation. Serious air leaks related to HHFNC therapy have been reported in pediatric patients [6]. Our case was an adult patient; however, her background status, which means post-ARDS, seemed to predispose to air leaks. In this case, we think from the image findings that air may initially leak into the mediastinum and the subcutaneous tissue, and then into a part of pleural space.

The reason why the pneumothorax was localized is probably because her pleural space was adhesive after inflammatory reaction. After cessation of HHFNC therapy, airflow under spontaneous breathing without any artificial support was regulated in her airway because of the absence of excessive airway pressure, which did not worsen air leaks and improved her respiratory condition.

## Conclusion

Positive pressure ventilation should be used with caution if there is a possible airway injury. HHFNC also may contribute to the development of air leak syndrome although HHFNC therapy tends to be sought gentler than other conventional therapy.

## Data Availability

The authors have the availability of supporting data.
